# A Novel Intracardiac Echocardiography–Guided Approach for Vein of Marshall Ethanol Infusion in Persistent Atrial Fibrillation Ablation

**DOI:** 10.1155/cdr/9390824

**Published:** 2026-03-18

**Authors:** Xiang Huang, Xiao-Lan Li, Jin Feng, Wei Zhang, Xiao-Mei Li

**Affiliations:** ^1^ Department of Cardiology, Xiangyang No.1 People’s Hospital, Hubei University of Medicine, Xiangyang, China, hbmu.edu.cn; ^2^ Hubei Key Laboratory of Biological Targeted Therapy, Union Hospital, Tongji Medical College, Huazhong University of Science and Technology, Wuhan, China, hust.edu.cn

## Abstract

**Background:**

The vein of Marshall (VOM) ethanol infusion is an adjunctive strategy for persistent atrial fibrillation (AF) ablation, traditionally guided by VOM venography. However, venography does not allow real‐time evaluation of ethanol distribution and carries procedural risks. Intracardiac echocardiography (ICE) provides high‐resolution visualization for precise guidance but remains understudied clinically. This study was aimed at evaluating the feasibility, efficacy, and safety of a novel ICE‐guided VOM ethanol infusion.

**Methods:**

A retrospective, matched cohort study was conducted including 126 patients (42 ICE‐guided, 84 venography‐guided) undergoing de novo radiofrequency (RF) catheter ablation for persistent AF. Propensity score matching (1:2) balanced the groups′ baseline characteristics. Procedural endpoints included ethanol‐induced low‐voltage area (LVA), procedural efficiency (procedure duration and radiation exposure), complications, and 12‐month AF/atrial tachycardia (AT) recurrence. ICE‐guided procedures utilized real‐time monitoring of ethanol‐induced tissue changes, whereas venography relied on contrast extravasation.

**Results:**

ICE guidance produced significantly larger ethanol‐induced LVAs, shorter total procedure times, reduced fluoroscopy times, shorter VOM ethanol infusion times, shorter mitral isthmus (MI) ablation times, and lower radiation exposure (all *p* < 0.05). Major complications were rare and similar between groups (*p* = 0.719). At 12 months, freedom from AF/AT recurrence was comparable between groups (log‐rank test, *p* = 0.66).

**Conclusions:**

ICE‐guided VOM ethanol infusion enhances procedural precision, reduces radiation exposure, and achieves more extensive substrate modification compared with venography. Although clinical recurrence rates were similar, ICE improves workflow efficiency and improves safety by avoiding distal VOM cannulation. These findings support ICE as a transformative tool for optimizing VOM ethanol ablation. Limitations include the nonrandomized design and small sample size, which warrant further validation in randomized trials.

## 1. Introduction

The ligament of Marshall (LOM), originating from the embryological remnant of the left superior vena cava, is positioned epicardially along the left lateral ridge, situated between the left pulmonary veins (LPVs) and the left atrial appendage (LAA) [1]. Its proximal segment is anatomically connected to the myocardial sleeves surrounding the coronary sinus (CS), while the distal portion extends superiorly toward the left atrial (LA) free wall or the pulmonary veins (PVs). The LOM contains various structures, including the VOM, myocardial sleeves, autonomic nerves, and ganglia [1, 2]. The VOM, which drains into the proximal CS, is the core structure of the LOM [3]. Ethanol infusion into the VOM can eliminate epicardial connections through the LOM, facilitate pulmonary vein antrum isolation (PVAI), and aid in MI ablation. [4, 5] Traditionally, VOM ethanol infusion has been guided by repeated venography [6]. However, venography only displays contrast extravasation and lacks further evaluative capabilities. This limitation can result in incomplete ablation with anhydrous ethanol, thereby reducing procedural efficacy [ 7].

ICE provides real‐time, high‐resolution visualization of cardiac anatomical structures. It precisely guides catheter positioning, dynamically assesses tissue contact during ablation, and promptly identifies anatomical variations or procedural complications. This optimizes procedural safety and efficacy, making ICE an essential adjunctive tool for AF ablation procedures [8, 9]. Ding et al. [10] first reported ICE‐guided VOM ethanol infusion, demonstrating consistent ethanol‐induced ablation effects along the LAA‐LSPV ridge. This approach increased the ablated tissue volume and reduced RF catheter ablation time. Despite these important clinical implications, ICE‐guided VOM ethanol infusion remains underevaluated in clinical practice.

This study retrospectively compared the feasibility and efficacy of VOM ethanol infusion under ICE guidance versus traditional venography guidance using a matched cohort design. Additionally, the procedural workflow associated with this technique was further optimized.

## 2. Materials and Methods

### 2.1. Study Design

At Xiangyang No. 1 People′s Hospital in Hubei Province, China, medical records of patients who underwent their initial RF catheter ablation for symptomatic persistent AF were retrospectively reviewed from January 2022 to May 2024. The hospital houses a dedicated electrophysiology laboratory fully equipped for advanced cardiac procedures and serves as a specialized referral center. The study complied with the principles of the Declaration of Helsinki and was approved by the Ethics Committee of Xiangyang No. 1 People′s Hospital (Approval Number 2025KY030).

### 2.2. Study Population

Patients with symptomatic persistent AF who underwent their first RF catheter ablation and completed all follow‐up visits between January 2022 and May 2024 were retrospectively enrolled. All procedures were performed by two experienced operators proficient in both VOM ethanol infusion and ICE. The study was aimed at comparing the efficacy and safety of two VOM ethanol infusion guidance modalities: ICE‐guided (group ICE, *n* = 42) versus venography‐guided (group venography, *n* = 84). Propensity score matching (PSM) was employed at a 1:2 ratio to match 42 ICE‐guided patients with 84 venography‐guided patients.

Patients who met the eligibility criteria for RF ablation due to persistent AF were included in the analysis [11]. Exclusion criteria included (1) age < 18 or > 80 years, (2) severe uncontrolled hypertension, (3) existing bradycardia or a history of pacemaker implantation, (4) significant hepatic dysfunction (hepatic biomarkers > 5 × the normal upper limit) or renal impairment (estimated glomerular filtration rate ≤ 60 mL/min/1.73 m^2^), (5) AF duration < 1 week or > 3 years, (6) left atrial diameter (LAD) > 65 mm or volume > 200 mL, (7) left ventricular ejection fraction (LVEF) < 35*%* or New York Heart Association (NYHA) Class III or IV heart failure, (8) ICE not utilized during the procedure, (9) absence of the VOM, (10) CS dissection or inability to infuse ethanol into the VOM, (11) structural heart disease except for left ventricular hypertrophy, (12) terminal illness with a life expectancy < 1 year, (13) incomplete follow‐up, and (14) other conditions deemed inappropriate for inclusion by the investigators.

### 2.3. Intraprocedural Management

Transesophageal echocardiography (TEE) was performed either 24 h before the procedure or ICE was used intraoperatively to rule out the presence of LA or LAA thrombus. Direct oral anticoagulants (DOACs) were administered without interruption during procedures performed under local anesthesia with deep sedation. The activated clotting time (ACT) was maintained between 300 and 350 s after femoral venous access using intravenous heparin (100 U/kg bolus, followed by 15 U/kg/h). A 6‐Fr decapolar electrophysiology catheter (IBI, St. Jude Medical) was positioned in the CS. Transseptal puncture (BRK Transseptal Needle, Abbott Medical) was performed under ICE guidance (SOUNDSTAR Ultrasound Catheter, Biosense Webster). An 8.5‐Fr nonsteerable sheath (SL1, Abbott Medical) was advanced into the LA, with an additional steerable sheath (Agilis NxT, Abbott Medical) used as necessary. Prior to ethanol infusion into the VOM, three‐dimensional anatomical reconstruction, along with detailed activation and voltage mapping of the LA and PVs, was conducted using a high‐density multipolar mapping catheter (Pentaray NAV ECO, Biosense Webster) integrated with a 3D electroanatomic mapping system (Carto 3, Biosense Webster).

### 2.4. Venography‐Guided VOM Ethanol Infusion Procedure

The CS was initially accessed through an SL1 sheath using a 6‐Fr guiding catheter (Judkins R 4.0/3.5, Cordis). Occasionally, an 8.5‐Fr steerable sheath was used. To locate the VOM, the guiding catheter was employed to perform nonocclusive venography of the CS in either the right anterior oblique (RAO) or left anterior oblique (LAO) projection. The VOM typically arises from a CS branch directed posteriorly or superiorly. Using the guiding catheter, selective cannulation was accomplished after identifying the VOM. Subsequently, an angioplasty guidewire (Runthrough NS Extra Floppy, Terumo) was advanced into the VOM, followed by placement of an over‐the‐wire (OTW) balloon catheter (length 8–12 mm, diameter 1.5–2.5 mm; Boston Scientific). Balloon inflation (maximum pressure: 6–8 atm) was performed distally within the VOM to achieve vessel occlusion, verified by injecting 0.5–1 mL of contrast through the balloon lumen (after guidewire removal). Following confirmation of complete occlusion and clear anatomical visualization, 95% ethanol was infused slowly (approximately 1 mL/min) at each balloon position. In the venography‐guided group, infusion was titrated according to venographic and safety endpoints. Ethanol infusion at a given position was ceased upon meeting any of the following: (1) Clear and homogeneous myocardial staining of the VOM territory was observed, and no further expansion of the staining occurred with additional small test injections; (2) contrast extravasation, vessel dissection, or a sudden increase in injection resistance suggesting high intraluminal pressure was noted; (3) a maximum of 4‐mL ethanol had been delivered at that position. Ethanol delivery was repeated sequentially at two to three positions within the VOM, from distal to proximal, separated by intervals of at least 2 min. The total ethanol volume for all positions was capped at 12 mL (three positions × up to 4 mL), which served as an upper safety cap but was not a mandatory target dose. Visualization of localized myocardial staining along the VOM indicated successful ethanol delivery. Postinfusion voltage mapping was repeated to assess ethanol‐induced LVAs.

### 2.5. ICE‐Guided VOM Ethanol Infusion Procedure

**Figure 1:**
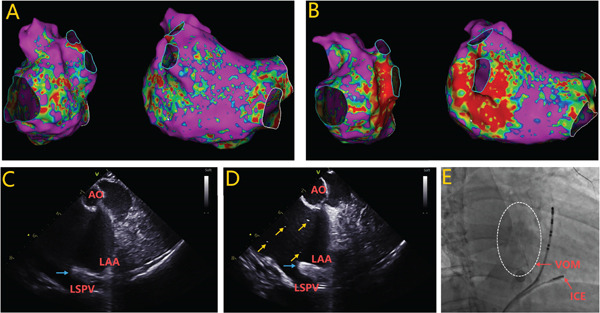
ICE‐guided VOM ethanol infusion. (a, b) Voltage mapping of the LA before and after ethanol infusion demonstrating new ethanol‐induced LVAs at the LAA‐LSPV ridge, posterior LA wall, and the MI‐adjacent region of the LIPV. (c, d) Real‐time ICE monitoring revealed dynamic morphological changes during VOM ethanol infusion, characterized by the progressive transformation of the LAA‐LSPV ridge from hypoechogenicity to hyperechogenicity (blue arrows) with consistent echogenic streaming (yellow arrows) in the LA. (e) The ICE catheter was placed in the right ventricle adjacent to the base of the aorta, with its tip facing anterosuperiorly toward the LAA. VOM venography showed myocardial staining visualized along the epicardial course of the VOM. The echocardiography‐enhanced area, ethanol‐induced LVA, and contrast myocardial staining area of the LA exhibited correlation. AO = aorta; ICE = intracardiac echocardiography; LA = left atrium; LAA = left atrial appendage; LIPV = left inferior pulmonary vein; LV = left ventricle; MI = mitral isthmus; PV = pulmonary vein; RA = right atrium; RV = right ventricle; VOM = vein of Marshall.

The main procedural differences between ICE‐guided and venography‐guided approaches involved balloon positioning and monitoring methods. In the ICE‐guided group, the OTW balloon was positioned at the VOM orifice without distal advancement. Balloon occlusion was angiographically confirmed via VOM venography. Ethanol infusion volumes were limited to 12 mL, administered slowly at 1–2 mL/min. To monitor lesion development along the ridge and echogenic streaming patterns within the LA in real time, an 8‐Fr ICE catheter (SOUNDSTAR Ultrasound Catheter, Biosense Webster) was inserted into the right femoral vein prior to ethanol administration. ICE images were primarily obtained through the right ventricular (RV) view of the lateral LA. From the RV view, the ICE catheter was gently advanced into the RV. By rotating clockwise from the RV position, the aortic root could be optimally visualized in the short‐axis view, enabling clear visualization of the lateral LA ridge between the LAA‐LSPV ridge (Figure 1a,b). The procedural endpoint was defined as a > 50% increase in tissue echogenicity at the LAA‐LSPV ridge, with or without accompanying echogenic streaming (Supporting Information). This endpoint was assessed qualitatively in real time by two experienced operators under standardized ICE settings (fixed depth, gain, dynamic range, and transducer frequency). The “> 50% increase” criterion was defined as a visually apparent change in which at least half of the atrial wall at the LAA‐LSPV ridge became distinctly hyperechogenic, compared with its relatively hypoechogenic state in the baseline image acquired immediately before ethanol infusion (Figure 1c). In defining this criterion, we relied on clinical experience and its consistency with successful ablation outcomes [10]. In cases of uncertainty, the final judgment was reached by consensus. No automated quantitative grayscale analysis was performed. Ethanol infusion ceased upon reaching a cumulative volume of 12 mL, regardless of echocardiographic enhancement status, and voltage mapping was also repeated after infusion to assess ethanol‐induced LVAs (Figure 1d,e).

### 2.6. RF Catheter Ablation Procedure

A 3.5‐mm irrigated‐tip ablation catheter with contact‐force detection (Thermocool SmartTouch, Biosense Webster) was used for RF energy delivery. The power‐control mode was commonly used to deliver ablation lesions, typically utilizing 30–40 W within the LA and maintaining temperatures below 43°C. The posterior wall of the LA, which is adjacent to the esophagus, was treated with lower power (20–25 W). RF lesion markers were automatically annotated with Carto VisiTag (Biosense Webster), applying filter thresholds: catheter movement < 2.0 mm within 3 s and stable contact force ≥ 8 g during ≥ 70% of lesion delivery duration.

Following VOM ethanol infusion, all patients underwent RF ablation using the “2C3L” strategy (two circumferential PVAIs and three linear ablations), guided by ablation index (AI) (Carto 3 System, Biosense Webster) targets [12, 13]. Detailed procedures were previously documented as follows: (1) circumferential pulmonary vein antrum (PVA) ablation with AI targets: 500–550 (anterior wall), 350–400 (posterior wall), reduced to 250–350 near the esophagus; [2] LA roof line (AI 450–500) connecting bilateral PVA lesions; [3] linear ablation along the MI was performed from the left inferior PV to the lateral mitral annulus (target AI 550–600), with supplemental epicardial ablation within the CS performed when necessary; and [4] additionally, cavotricuspid isthmus (CTI) ablation (AI 450–500) extended from the inferior tricuspid annulus (approximately 6 o′clock position) to the inferior vena cava.

In cases where achieving MI block endocardially was challenging post‐VOM ethanol infusion, combined epicardial–endocardial ablation was utilized. Additional ablations, such as LA posterior wall isolation (box lesion) or superior vena cava isolation, were performed at the operator′s discretion based on procedural outcomes. Electrical cardioversion was performed when AF persisted after ablation. ICE was used routinely before and after ethanol infusion and at procedure completion to evaluate for pericardial effusion around the posterior LA and inferior cardiac border.

### 2.7. Postprocedure Treatments and Follow‐Up

All patients were discharged on antiarrhythmic drugs (AADs) and oral anticoagulants (OACs). AADs were discontinued 3 months postablation if patients remained free from recurrent atrial tachyarrhythmias. Long‐term OAC use was recommended based on thromboembolic risk evaluation following current guidelines. Patients were followed up by telephone and/or in the outpatient clinic once a month after ablation. At 3, 6, 9, and 12 months after ablation, or earlier if symptoms appeared, patients were scheduled for transthoracic echocardiography and 24‐h Holter monitoring as part of their follow‐up assessments. Any documented episode of AF, atrial flutter, or AT lasting more than 30 s following a blanking interval of 3 months was considered a clinical recurrence. The major endpoint of the trial was the absence of AF or atrial tachyarrhythmia.

### 2.8. Statistical Analysis

To minimize selection bias and balance baseline characteristics between the ICE‐guided and venography‐guided groups, PSM was performed at a 1:2 ratio using the nearest neighbor matching algorithm without replacement. A caliper width of 0.2 of the standard deviation (SD) of the logit of the propensity score was applied to ensure match quality. The model included baseline demographic and clinical variables that were considered potentially associated with outcomes, incorporating the following covariates: age, sex, body mass index (BMI), LAD, LVEF, left ventricular internal diameter at end diastole (LVIDd), NYHA functional class, hypertension, diabetes mellitus, coronary artery disease, and history of stroke or transient ischemic attack (TIA). All matching procedures were conducted using the MatchIt package in R software (Version 4.5.0). All 42 patients in the ICE‐guided group were successfully matched. The adequacy of matching was confirmed by the absence of significant differences in all included covariates between the matched groups (all *p* > 0.05, Table 1). Following the initial observations, further investigations were conducted on the matched patient cohorts.

**Table 1:** Baseline characteristics.

	Unmatched sample			Matched sample		
	Group ICE (*n* = 42)	Group venography (*n* = 219)	*p*	Group ICE (*n* = 42)	Group venography (*n* = 84)	*p*
Age (years)	67.5 (55, 74)	65 (58, 73)	0.806	67.5 (55, 74)	67.5 (56, 73)	0.930
Male (%)	19 (45.2)	111 (50.7)	0.518	19 (45.2)	39 (46.4)	0.899
BMI (kg/m^2^)	22.9 ± 3.15	22.7 ± 3.87	0.762	22.9 ± 3.15	22.8 ± 3.81	0.867
Hypertension	13 (31.0)	78 (35.6)	0.561	13 (31.0)	28 (33.3)	0.788
CAD	5 (11.9)	20 (9.1)	0.576	5 (11.9)	7 (8.3)	0.520
Diabetes mellitus	7 (16.7)	66 (30.1)	0.075	7 (16.7)	25 (29.8)	0.111
NYHA > II (%)	16 (38.1)	77 (35.2)	0.716	16 (38.1)	30 (35.7)	0.794
Stroke/TIA	3 (7.1)	25 (11.4)	0.412	3 (7.1)	5 (6.0)	0.796
LVEF (%)	51 (48, 56)	51 (50, 56)	0.680	51 (48, 56)	51 (46.5, 55)	0.769
LAD (mm)	49 (38, 50)	41 (38, 48)	0.031	49 (38, 50)	43 (38.5, 52)	0.771
LVIDd (mm)	47 (43, 55)	44 (42, 51)	0.009	47 (43, 55)	48 (42, 51)	0.261
CHA_2_DS_2_‐VASc score	2 (2, 3)	2 (2, 3)	0.255	2 (2, 3)	2 (2, 2)	0.469

Abbreviations: BMI, body mass index; CAD, coronary artery disease; LAD, left atrial diameter; LVEF, left ventricular ejection fraction; LVIDd, left ventricular internal diameter at end diastole; NYHA, New York Heart Association; TIA, transient ischemic attack.

Variance homogeneity was confirmed by Levene′s test, and the normality of continuous data was evaluated using the Shapiro–Wilk test. Continuous variables with normal distribution were reported as mean ± SD, whereas nonnormally distributed variables were presented as medians (interquartile range [IQR]). Comparisons between independent groups were performed using independent samples *t*‐tests (with Welch′s correction when necessary) or Mann–Whitney *U* tests. Categorical data were summarized as frequencies and percentages and analyzed using Pearson′s chi‐square or Fisher′s exact tests as applicable. Kaplan–Meier survival analyses with log‐rank tests compared recurrence‐free survival between groups. A two‐tailed *p* value < 0.05 was considered statistically significant. All statistical analyses were conducted using SPSS software (Version 26.0, IBM Corp.).

## 3. Results

### 3.1. Baseline Characteristics

**Figure 2:**
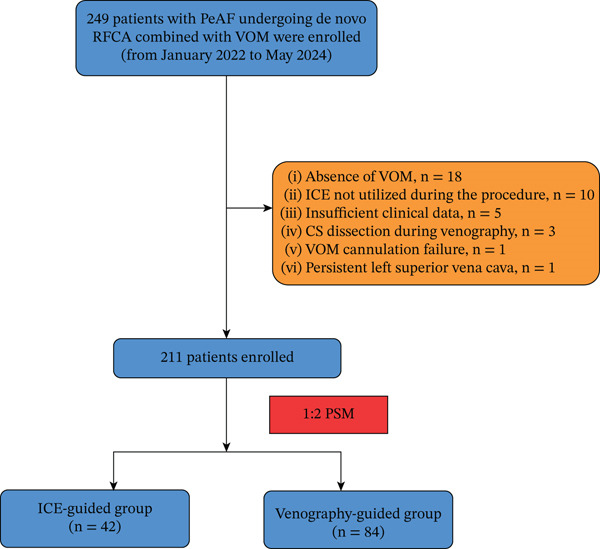
Study flow. PeAF = persistent atrial fibrillation; RFCA = radiofrequency catheter ablation; VOM = vein of Marshall; ICE = intracardiac echocardiography; CS = coronary sinus; PSM = propensity score matching.

A total of 249 patients diagnosed with persistent AF who underwent RF catheter ablation combined with VOM ethanol infusion were initially assessed. Among these, 24 patients (9.6%) were excluded due to the inability to perform VOM ethanol infusion, specifically 18 patients without a discernible VOM, 3 patients who underwent CS dissection during venography, 1 patient with VOM cannulation failure attributed to a small vessel diameter, and 1 patient with a persistent left superior vena cava. Other exclusions included 10 patients unable to receive ICE guidance due to financial constraints and 5 patients with insufficient clinical data. In total, the analysis comprised 211 patients. PSM at a 1:2 ratio was conducted, resulting in the pairing of 42 patients who underwent ICE‐guided ethanol infusion with 84 patients who received venography‐guided procedures (Figure 2). Table 1 presents the baseline demographic and clinical characteristics.

### 3.2. Early Procedural Outcomes

After matching, acute MI block was similar between groups: Acute MI block was achieved in 42 patients (100%) in the ICE‐guided group and 78 patients (92.86%) in the venography‐guided group (Table 2). The total ethanol consumption volume was 8 mL (6–9 mL) in the ICE‐guided group versus 7 mL (5–8 mL) in the venography‐guided group; while this difference did not reach statistical significance (*p* = 0.059, Table 2), we acknowledge the numerical trend. However, ethanol‐induced LVAs were significantly larger (7.07 cm^2^ [5.14–9.97 cm^2^] in the ICE‐guided group vs. 3.62 cm^2^ [2.20–5.71 cm^2^] in the venography‐guided group; *p* < 0.001, Table 2). Additionally, 38 patients (90.5%) in the ICE‐guided group demonstrated echocardiography‐enhanced hydropic myocardium with or without echogenic streaming during infusion.

**Table 2:** Procedural data and electrophysiological findings.

	Group ICE (*n* = 42)	Group venography (*n* = 84)	*p*
Total procedure time (min)	129.45 (120.8, 140.6)	142.4 (130.4, 185.8)	< 0.001
Fluoroscopy time (min)	7.65 (4.6, 9.8)	10.35 (8.2, 11.7)	< 0.001
EI‐VOM time (min)	10.5 (9.1, 13.1)	16.35 (12.45, 20.25)	< 0.001
PVAI time (min)	16.0 (14.9, 24.7)	17.95 (15.05, 25.0)	0.559
MI ablation time (min)	5.9 (4.6, 7.9)	7.3 (4.65, 10.5)	0.042
Roofline ablation time (min)	3.6 (2.2, 5.1)	4.1 (3.05, 5.15)	0.233
CTI ablation time (min)	5.4 (3.2, 7.1)	5.6 (3.65, 6.95)	0.621
Ethanol consumption (mL)	8 (6, 9)	7 (5, 8)	0.059
Radiation exposure (mGy)	53.5 (35.5, 123)	130 (120, 146)	< 0.001
PVAI (%)	42 (100)	84 (100)	—
Linear block			
MI (%)	42 (100.00)	78 (92.86)	0.076
CTI (%)	42 (100)	83 (98.8)	0.478
Roofline (%)	42 (100)	84 (100)	—
Ablation in the CS (%)	12 (28.6)	27 (32.9)	0.683
LA posterior wall isolation (%)	7 (16.7)	10 (11.9)	0.461
SVC isolation (%)	4 (9.5)	9 (10.7)	0.836
Electrical cardioversion (%)	31 (73.8)	66 (78.6)	0.549
LVA			
LVA before EI‐VOM (cm^2^)	3.44 (2.66, 4.33)	3.66 (2.58, 4.65)	0.632
LVA after EI‐VOM (cm^2^)	11.35 (8.80, 13.94)	8.08 (6.27, 9.51)	< 0.001
*Δ*LVA (cm^2^)	7.07 (5.14, 9.97)	3.62 (2.20, 5.71)	< 0.001
Complications			
Pericardial effusion (%)	1 (2.4)	3 (3.6)	0.719
Death (%)	0	0	—
Stroke (%)	0	0	—
Atrial–esophageal fistula (%)	0	0	—

Abbreviations: *Δ*LVA, differences in LVA before and after ethanol infusion; CS, coronary sinus; CTI, cavotricuspid isthmus; EI‐VOM, vein of Marshall ethanol infusion; LA, left atrial; LVA, low‐voltage area; MI, mitral isthmus; PVAI, pulmonary vein antrum isolation; SVC, superior vena cava.

On the premise that the distribution of other additional ablation strategies was similar between the groups (*p* > 0.05, Table 2), procedural efficiency was significantly enhanced in the ICE‐guided group, with significantly shorter total procedure time (129.45 min [120.8, 140.6] vs. 142.4 min [130.4, 185.8], *p* < 0 0.001, Table 2), shorter fluoroscopy time (7.65 min [4.6, 9.8] vs. 10.35 min [8.2, 11.7], *p* < 0 0.001, Table 2), and shorter VOM ethanol infusion time (10.5 min [9.1, 13.1] vs. 16.35 min [12.45, 20.25], *p* < 0 0.001, Table 2) and MI ablation time (5.9 min [4.6, 7.9] vs. 7.3 min [4.65, 10.5], *p* = 0 0.04, Table 2) recorded in the ICE‐guided group compared with the venography‐guided group. And less radiation exposure (53.5 mGy [35.5, 123] vs. 130.0 mGy [120, 146], *p* < 0 0.001, Table 2) was recorded in the ICE‐guided group.

### 3.3. Procedure‐Related Complications and Follow‐Up

Major complication rates did not differ significantly between groups. In the venography‐guided group, three patients (3.6%) developed pericardial effusion during the procedure, including one patient (1.2%) who required pericardiocentesis. In the ICE‐guided group, one patient (2.4%) experienced transient pericardial effusion that resolved spontaneously. No severe complications (death, stroke, or atrioesophageal fistula) occurred. Although complications numerically favored ICE guidance (*p* = 0.719; Table 2), overall rates remained comparable.

**Figure 3:**
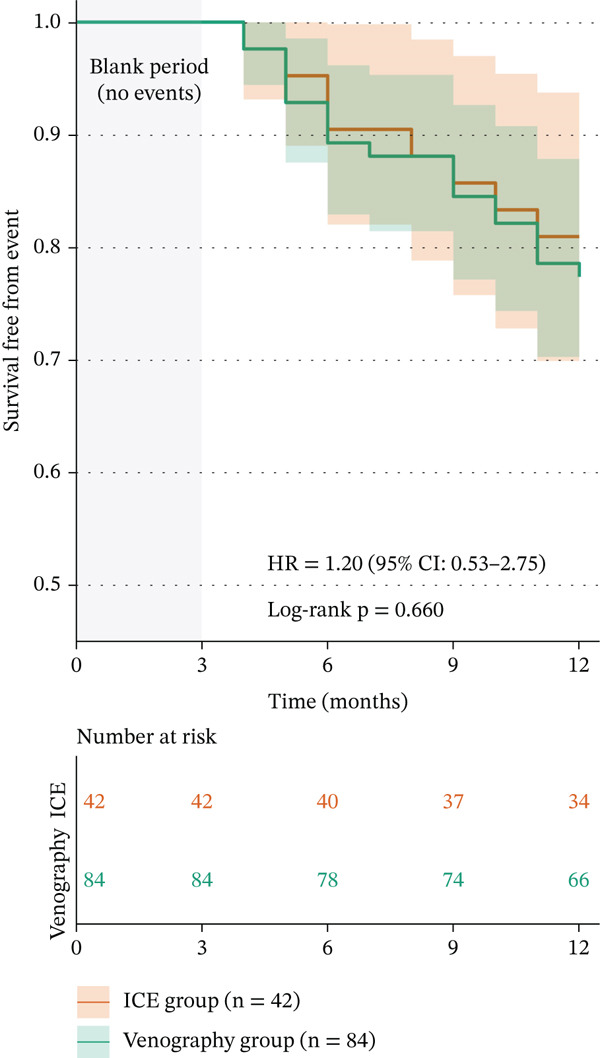
Kaplan–Meier curve showing survival free from AF/AT recurrence with or without AADs in both groups. AADs = antiarrhythmic drugs; AF = atrial fibrillation; AT = atrial tachycardia.

At the 12‐month follow‐up, 34 (80.95%) patients were free from AF/AT in the ICE‐guided group compared with 66 (78.57%) in the venography‐guided group. Among them, 8 patients in the ICE‐guided group and 12 patients in the venography‐guided group were on AADs during the 12‐month follow‐up (*p* = 0.491). Using recurrence of AF or AT as the dependent variable and group assignment as the independent variable, Kaplan–Meier analysis suggested that the venography‐guided group was associated with recurrence; however, this association did not reach statistical significance (hazard ratio: 1.20; 95% confidence interval: 0.53–2.75; log‐rank test *p* = 0.66) (Figure 3).

## 4. Discussion

Recent evidence highlights the important role of the VOM in atrial arrhythmogenesis due to its dual autonomic denervation (sympathetic and parasympathetic), direct electrical connections that facilitate focal or reentrant activity, and anatomical proximity to the MI, contributing to atrial flutter and fibrillation maintenance [14, 15]. Targeting these arrhythmogenic substrates with ethanol induces autonomic denervation and disrupts residual endo–epicardial electrical connections, thereby improving MI block efficacy and reducing arrhythmia recurrence [16]. However, the VOM exhibits significant anatomical variability in luminal diameter, length, and morphology among individuals [17]. Venography‐guided VOM ethanol infusion has several limitations: (1) Advancing guidewires into the distal VOM, where luminal diameter narrows, increases the risk of perforation; (2) OTW balloon dilation in the distal VOM may cause vascular dissection; (3) venography visualizes only contrast extravasation around the VOM during ethanol infusion, lacking a clear and objective ablation endpoint, potentially causing suboptimal ethanol delivery and reduced therapeutic efficacy.

This study assessed the practicality and clinical efficacy of ICE‐guided VOM ethanol infusion. As described in the Methods, the ICE catheter was positioned to obtain the RV view. From this orientation, clockwise rotation of the catheter provided a clear imaging window to the LAA‐LSPV ridge (Figure 1c,e). During ethanol infusion, we dynamically monitored two key phenomena: (1) Focal tissue echogenicity enhancement—the LAA‐LSPV ridge transitioned from a relatively hypoechoic state to a diffusely hyperechoic appearance (Figure 1c,d, blue arrows), corresponding to ethanol‐induced tissue edema and acute injury. The procedural endpoint was defined as a > 50% qualitative increase in echogenicity, characterized by at least half of the target atrial wall becoming distinctly hyperechogenic relative to baseline. (2) Echogenic streaming—the appearance and flow of hyperechoic microbubbles or particulate matter within the LA cavity (Figure 1c,d, yellow arrows), suggesting ethanol entry into the atrial cavity via intact venoatrial microcirculatory connections.

Notably, the two guidance approaches (ICE vs. venography) are consistent in all procedural aspects except for the monitoring and guidance method during VOM ethanol infusion, including patient selection, intraprocedural management, RF ablation strategy (“2C3L” approach), postprocedural care, and follow‐up protocols. This consistency ensures that observed differences are specifically attributed to the VOM ethanol infusion guidance modality.

Our findings demonstrate that ICE provides greater precision and real‐time visualization of ethanol infusion effects compared with VOM venography, offering critical guidance for determining ablation endpoints. Furthermore, ICE‐guided VOM ethanol infusion fundamentally altered the procedural rationale by providing a real‐time tissue endpoint, thereby obviating the routine need for guidewire and OTW balloon advancement into the distal VOM and streamlining workflow efficiency. This key modification confers safety benefits: The distal VOM is often narrow and tortuous, so advancing guidewires/balloons in venography‐guided procedures increases risks of vascular perforation, dissection, and subsequent pericardial effusion. By positioning the balloon only at the VOM orifice and relying on real‐time echogenicity changes and echogenic streaming to confirm efficacy, ICE‐guided procedures completely avoid these distal manipulation‐related hazards. Although proximal balloon positioning is achievable under venographic guidance, the lack of real‐time feedback often drives operators to default to distal advancement as an empirical strategy to ensure efficacy—a maneuver that inherently increases procedural risk. By contrast, the visual confirmation of tissue effects provided by ICE enables operators to confidently achieve effective ablation from a proximal position.

In our study, ICE‐guided VOM ethanol infusion reduced reliance on fluoroscopy, minimized radiation exposure, and avoided complications such as guidewire perforation or balloon‐induced vascular dissection by eliminating distal VOM cannulation. Crucially, this approach established a more objective and reproducible procedural endpoint. Ethanol‐induced LVAs reflect the extent of myocardial injury and correlate with procedural success [18]. Our ICE‐guided group demonstrated larger ethanol‐induced LVAs and shorter procedural durations (total procedure time, VOM ethanol infusion time, and MI ablation time). This suggests ICE‐guided ethanol infusion achieves more extensive substrate modification, decreasing reliance on adjunctive RF ablation. In contrast, the venography‐guided approach, lacking real‐time tissue feedback, often relied on reaching a pre‐emptive safety limit (volume or staining), potentially leading to underinfusion before an optimal ablative effect was achieved. Therefore, ICE guidance allowed for more complete and effective substrate modification within a similarly safe dosage range, as evidenced by significantly larger LVAs achieved despite a nonsignificant trend toward higher ethanol consumption volume. Furthermore, this study provides objective validation for the ICE‐guided procedural endpoint. Although the criterion of a > 50% increase in echogenicity was applied as a standardized, qualitative visual assessment by experienced operators, it was rigorously corroborated by a consistent and significant association with the formation of ethanol‐induced LVAs on detailed electroanatomic mapping. This indicates that the ICE‐derived endpoint, while experience‐based at the time of acquisition, functions as a visually intuitive marker that reliably predicts definitive electrophysiological substrate modification.

Additionally, VOM ethanol infusion is aimed at enabling ethanol to retrogradely penetrate LA tissue through the cardiac venous circulation. Therefore, ethanol should be injected slowly under controlled pressure, overcoming anterograde flow into LA tissue while avoiding excessive intravascular pressure, which could cause venular rupture or phrenic nerve injury. [19] During infusion, ICE‐detected echogenic streaming in the LA reflects capillary‐mediated connectivity among VOM branches, atrial tissue, and the LA cavity. [20] Consistent streaming indicates intact venules and assists in assessing appropriate injection pressure.

## 5. Conclusion

ICE represents a transformative tool for VOM ethanol ablation in persistent AF. Its ability to provide real‐time tissue feedback improves procedural accuracy, reduces total ablation time, and minimizes radiation exposure. Compared with conventional VOM venography, ICE‐guided VOM ethanol ablation optimizes MI ablation through improved lesion completeness, as demonstrated by significantly larger LVAs and shorter MI RF ablation duration. Future research should explore integrating ICE with advanced mapping systems and evaluate long‐term outcomes to refine its clinical role.

## 6. Study Limitations

Despite encouraging results, this study is limited by its nonrandomized design and small sample size. Furthermore, all ICE‐guided procedures were conducted between January and May 2024, and variability in operator proficiency during this period may introduce bias. Additionally, real‐time ICE‐detected tissue changes occurred in only 90.5% of ICE‐guided VOM ethanol infusion procedures. Possible explanations include limitations of two‐dimensional ultrasound imaging in visualizing enlarged LA anatomies and ethanol‐induced venule rupture. Venule rupture, frequently visualized as localized contrast leakage due to mechanical factors, may also lead to pericardial effusion [7, 21]. Future refinements of this ICE‐guided approach could involve the integration of quantitative ultrasound software for more objective, pixel intensity–based measurement of echogenicity changes, further standardizing the endpoint assessment.

A prospective study design directly comparing tissue echogenicity at the LAA‐LSPV ridge in ICE‐guided versus venography‐guided groups upon achieving predefined ablation endpoints would further validate the utility of ICE for optimizing VOM ethanol infusion. This comparison could clarify whether ICE‐specific visualization of hydropic myocardium and streaming directly correlates with improved lesion efficacy or long‐term outcomes.

## Author Contributions

Xiang Huang, Wei Zhang, Jin Feng, and Xiao‐Mei Li conceived and designed the study. Xiao‐Lan Li acquired data and interpreted the data. Xiang Huang and Xiao‐Mei Li served as the primary operators for the procedure. Wei Zhang has addressed the reviewers′ comments along with the edits to the supporting information video file. Wei Zhang and Xiao‐Mei Li contributed equally to this study and shared the correspondence authorship.

## Funding

This study was supported by the Open Foundation of Hubei Key Laboratory of Biological Targeted Therapy (202410).

## Disclosure

All the authors critically revised the manuscript for important intellectual content.

## Ethics Statement

The present study was approved by the Xiangyang No. 1 People′s Hospital Ethics Committee and adhered to the tenets of the Declaration of Helsinki (Approval Number 2025KY030). Owing to the retrospective nature of the present study, written informed consent from the patients or their guardians was waived.

## Conflicts of Interest

The authors declare no conflicts of interest.

## Supporting information


**Supporting Information To** Additional supporting information can be found online in the Supporting Information section. Further illustrate the relevant findings, video files in the supporting information demonstrate the dynamic evolution of imaging of the LAA‐LSPV ridge observed via ICE during VOM ethanol infusion.

## Data Availability

Data is available on reasonable request from the corresponding authors.
